# Inhibition of α-, β-, γ-, and δ-carbonic anhydrases from bacteria and diatoms with *N′*-aryl-*N*-hydroxy-ureas

**DOI:** 10.1080/14756366.2018.1490733

**Published:** 2018-07-25

**Authors:** Emanuela Berrino, Murat Bozdag, Sonia Del Prete, Fatmah A. S. Alasmary, Linah S. Alqahtani, Zeid AlOthman, Clemente Capasso, Claudiu T. Supuran

**Affiliations:** a Dipartimento Neurofarba, Sezione di Scienze Farmaceutiche e Nutraceutiche, Università degli Studi di Firenze, Florence, Italy;; b CNR, Istituto di Bioscienze e Biorisorse, Napoli, Italy;; c Department of Chemistry, College of Science, King Saud University, Riyadh, Saudi Arabia;; d Department of Chemistry, King Faisal University, Alahsa, Saudi Arabia

**Keywords:** Carbonic anhydrase, metalloenzymes, protozoa, activators, *Plasmodium falciparum*

## Abstract

The inhibition of α-, β-, γ-, and δ-class carbonic anhydrases (CAs, EC 4.2.1.1) from bacteria (*Vibrio cholerae* and *Porphyromonas gingivalis*) and diatoms (*Thalassiosira weissflogii*) with a panel of *N’*-aryl-*N*-hydroxy-ureas is reported. The α-/β-CAs from *V. cholerae* (VchCAα and VchCAβ) were effectively inhibited by some of these derivatives, with K_I_s in the range of 97.5 nM – 7.26 µM and 52.5 nM – 1.81 µM, respectively, whereas the γ-class enzyme VchCAγ was less sensitive to inhibition (K_I_s of 4.75 – 8.87 µM). The β-CA from the pathogenic bacterium *Porphyromonas gingivalis* (PgiCAβ) was not inhibited by these compounds (K_I_s > 10 µM) whereas the corresponding γ-class enzyme (PgiCAγ) was effectively inhibited (K_I_s of 59.8 nM – 6.42 µM). The δ-CA from the diatom *Thalassiosira weissflogii* (TweCAδ) showed effective inhibition with these derivatives (K_I_s of 33.3 nM – 8.74 µM). As most of these *N*-hydroxyureas are also ineffective as inhibitors of the human (h) widespread isoforms hCA I and II (K_I_s > 10 µM), this class of derivatives may lead to the development of CA inhibitors selective for bacterial/diatom enzymes over their human counterparts and thus to anti-infectives or agents with environmental applications.

## 1. Introduction

*N*-Hydroxyurea has been reported^1^ by our group as a new chemotype belonging to the family of inhibitors of the metallo-enzyme carbonic anhydrase (CA, EC 4.2.1.1)^2–6^. This simple compound has been shown to bind in an unprecedented manner to the metal ion from this enzyme active site (more precisely the human (h) isoform hCA II), by means of X-ray crystallographic and kinetic studies^1^. Although *N*-hydroxyurea is a weak, micromolar inhibitor, it was observed to coordinate bidentately to the Zn(II) ion from the hCA II active site, both through its NH and OH groups of the CONHOH fragment of the molecule (presumably deprotonated), which is rather unusual, as all the previously investigated inhibitors at that time were monodentate zinc ligands^2^. This discovery led to the detailed investigation of organic hydroxamates (RCONHOH) as CA inhibitors (CAIs), which are quite diverse from the main class, prototypical inhibitors of these enzymes, which are the sulfonamides and their isosteres, sulfamates, and sulfamides, all of them incorporating the SO_2_NH_2_ moiety as zinc-binding group (ZBG)^2–7^. Many representatives of these class of compounds, are in clinical use for decades, as they show diuretic^8^, antiglaucoma^9^, antiobesity^10^, antitumor^11^, anti-neuropathic pain^12^, and anti-arthritis^13^ effects. However, a main concern with sulfonamides/sulfamates/sulfamides as CAIs is their lack of selectivity for the many CA isoforms present in humans (15 different CAs belonging to the α-class)^3^. When considering all the CA families known to date (α-, β-, γ-, δ-, η-, ζ-, and θ-CAs) in organisms all over the phylogenetic tree^2–6^, the selectivity problem is really challenging, since sulfonamides and their derivatives generally act as effective inhibitors of enzymes belonging to all these diverse classes. Thus, the development of non-sulfonamide isoform- or class-selective CAIs is of great interest for targeting enzymes from parasitic bacteria, fungi, or protozoa, which in many cases contain non-α-CAs (which in turn are present in the vertebrate hosts, including humans, as mentioned above)^2–6^. Interesting developments have been reported in this field in recent years, in the search of anti-infectives with a new mechanism of action, devoid of the drug resistance problems encountered by many classes of antibiotic, antifungal, and anti-protozoan agents^4^^,^^5^. Indeed, some hydroxamates or carboxylates showed effective *in vitro* CA inhibitory properties and also anti-*Trypanosoma cruzi*, or anti-leishmanial activities *ex vivo*^4^. Thus, in the search of isoform- or class-selective CAIs we investigated here a class of recently developed *N*-hydroxy-ureas^14^, which incorporate a more elaborated organic scaffold attached to the second nitrogen atom (compared to the simple lead molecule, N-hydroxyurea)^1^ and which proved to be effective inhibitors of the tumor-associated isoforms hCA IX/XII^14^, without inhibiting considerably the off-target, house-keeping cytosolic isoforms hCA I and II, which are responsible for the many side effects seen with the sulfonamide type of CAIs^2–6^.

## 2. Materials and methods

### 2.1. Chemistry

Compounds **1–20** were prepared as reported earlier^14^. Buffers and acetazolamide (AAZ) were commercially available, highest purity reagents from Sigma-Aldrich/Merck, Milan, Italy.

### 2.2. CA enzyme inhibition assay

An Sx.18Mv-R Applied Photophysics (Oxford, UK) stopped-flow instrument has been used to assay the catalytic activity of various CA isozymes for CO_2_ hydration reaction^15^. Phenol red (at a concentration of 0.2 mM) was used as indicator, working at the absorbance maximum of 557 nm, with 10 mM HEPES (pH 7.5, for α- and δ-CAs) or tris (pH 8.3, for β- and γ-CAs) as buffers, 0.1 M sodium sulphate (for maintaining constant ionic strength), following the CA-catalyzed CO_2_ hydration reaction for a period of 10 s at 25 °C. The CO_2_ concentrations ranged from 1.7 to 17 mM for the determination of the kinetic parameters and inhibition constants. For each inhibitor at least six traces of the initial 5–10% of the reaction have been used for determining the initial velocity. The uncatalyzed rates were determined in the same manner and subtracted from the total observed rates. Stock solutions of inhibitors (10 mM) were prepared in distilled-deionized water and dilutions up to 1 nM were done thereafter with the assay buffer. Enzyme and inhibitor solutions were pre-incubated together for 15 min (standard assay at room temperature) prior to assay, in order to allow for the formation of the enzyme-inhibitor complex. The inhibition constants were obtained by non-linear least-squares methods using PRISM 3 and the Cheng-Prusoff equation, as reported earlier^14^^,^^16–22^. All CAs were recombinant proteins produced as reported earlier by our groups^16–22^.

## 3. Results and discussion

Bacterial, fungal, protozoan, or other organisms CAs may represent new drug targets for the development of anti-infectives with an alternative mechanism of action to clinically used agents, but this type of research was rather neglected for a long time^4^^,^^5^. Only in the last several years, mainly our group, cloned and investigated the inhibition of many parasite CAs from various organisms and belonging to a multitude of enzyme classes, providing the proof-of-concept experiments that parasite CA inhibitors may have a significant anti-infective effect, *in vitro* and *in vivo*, for many widespread pathogens such as those provoking malaria^4^^,^^5^, Chagas disease^4^^,^^5^, *Leishmania*^4^^,^^5^, or *Helicobacter pylori* infection^23^.

The rationale to investigate the new *N’*-aryl-*N*-hydroxyureas of compound type **1**–**20** as inhibitors of bacterial/diatom CAs, is based on the recent reported of Bozdag et al.^14^ that these compounds act as hCA IX/XII-selective inhibitors over hCA I and II (Table 1). In this article we included in the investigations the three CAs from the bacterial pathogen *Vibrio cholerae* (VchCAα/β/γ)^16^^,^^17^, the two CAs from the oral bacterial pathogen *Porphyromonas gingivalis* (PgiCAβ/γ)^18^^,^^24^ as well as the uniquely well investigated δ-class CA, TweCAδ, from the diatom *Thalassiosira weissflogii*^19^.

**Table 1.** Inhibition data of human CA isoforms hCA I, II, IX, and XII with Compounds **1**–**22** by a stopped flow CO_2_ hydrase assay^15^.


**1-20**				
K_I_ (nM)^a^				
Cmp	hCA I	hCA II	hCA IX	hCA XII
**1**	>10,000	>10,000	>10,000	27.4
**2**	>10,000	>10,000	>10,000	253.2
**3**	>10,000	>10,000	>10,000	>10,000
**4**	>10,000	>10,000	8237.3	491.2
**5**	>10,000	>10,000	>10,000	808.8
**6**	>10,000	>10,000	>10,000	>10,000
**7**	>10,000	>10,000	7781.7	43.6
**8**	>10,000	>10,000	>10,000	529.2
**9**	>10,000	>10,000	>10,000	>10,000
**10**	>10,000	>10,000	>10,000	768.0
**11**	>10,000	>10,000	>10,000	858.2
**12**	>10,000	>10,000	253.5	>10,000
**13**	>10,000	>10,000	679.1	27.9
**14**	>10,000	>10,000	78.9	7.2
**15**	>10,000	>10,000	>10,000	>10,000
**16**	>10,000	>10,000	>10,000	>10,000
**17**	>10,000	>10,000	268.9	51.3
**18**	>10,000	>10,000	130.0	42.1
**19**	>10,000	>10,000	>10,000	377.6
**20**	>10,000	>10,000	>10,000	746.6

a Mean from three different assays, by a stopped flow technique (errors were in the range of ±5–10% of the reported values)^14^.

Inhibition data of the six CAs mentioned above with Compounds **1–20** and acetazolamide (AAZ) as standard, sulfonamide inhibitor, are shown in Table 2. The following structure-activity relationship (SAR) is observed from thee data of Table 2:

**Table 2.** Inhibition of CAs belonging to the α-, β-, γ-, and δ-classes with *N*-hydroxyureas Compounds **1**–**20** and the standard sulfonamide inhibitor acetazolamide (AAZ), by a stopped-flow CO_2_ hydrase assay^15^.


**1-20**						
K_I_ (nM)^a^						
No: R	VchCAα	VchCAβ	VchCAγ	PgiCAβ	PgiCAγ	TweCAδ
**1:** H	7260	1810	>10,000	>10,000	6424	>10,000
**2:** 4-CH_3_	111.5	483	>10,000	>10,000	298	>10,000
**3:** 2-CH_3_	829	377	>10,000	>10,000	4214	>10,000
**4:** 4-Cl	4405	541	>10,000	>10,000	934	8740
**5:** 2-Cl	5941	64.2	>10,000	>10,000	3030	>10,000
**6:** 3-Cl	4000	60.3	>10,000	>10,000	819	>10,000
**7:** 4-O_2_N	509	54.1	>10,000	>10,000	2699	>10,000
**8:** 2-O_2_N	536	52.5	5687	>10,000	82.3	>10,000
**9:** 2,5-Me_2_	97.5	59.3	5500	>10,000	84.4	>10,000
**10:** 4-F	>10,000	>10,000	>10,000	>10,000	>10,000	>10,000
**11:** 3-EtOOC	>10,000	>10,000	>10,000	>10,000	>10,000	8490
**12:** 3,5-Me_2_	>10,000	>10,000	>10,000	>10,000	5493	57.8
**13:** 2-EtO	>10,000	>10,000	>10,000	>10,000	>10000	33.3
**14:** 3-MeS	>10,000	>10,000	>10,000	>10,000	59.8	52.0
**15:** 4-F-3-Me	>10,000	>10,000	>10,000	>10,000	2882	3935
**16:** F_5_	>10,000	>10,000	>10,000	>10,000	3482	3413
**17:** 4-CF_3_	>10,000	>10,000	>10,000	>10,000	2630	4640
**18:** 4-CF_3_-2-Cl	>10,000	>10,000	5426	>10,000	>10,000	856
**19:** 2-MeO	>10,000	>10,000	4750	>10,000	>10,000	2879
**20:** 4-PhO	>10,000	>10,000	>10,000	>10,000	>10,000	761
**AAZ**	6.8	451	473	214	324	83

^a^Mean from three different assays, by a stopped flow technique (errors were in the range of ±5–10% of the reported values, data not shown).

- VchCAα was inhibited by some but not all Compounds **1**–**20** with K_I_s in the range of 97.5 nM – 7.26 µM (Table 2). The best inhibitors were Compounds **2** and **9** (K_I_s of 111.5 and 97.5 nM, respectively) and both of them have a Me-Ph moiety in their molecule (Compound **9** has also a second methyl group). It seems that these two substitution patterns of the aromatic ring are particularly effective for inhibiting this enzyme. The nitro-containing derivatives (Compounds **7** and **8**), as well as the 2-Me derivative (Compound **3**) were the next best VchCAα inhibitors, with K_I_s < 1 µM, whereas the remaining derivatives (Compounds **1**–**6)** were weaker, micromolar inhibitors. Strangely enough, all Compounds **11**–**20** showed K_I_s > 10 µM, which proves that small changes in the substitution pattern at the aromatic ring has dramatic consequences for the CA inhibitory activity.
- VchCAβ showed a rather similar behavior, as Compounds **1**–**10** were effective inhibitors (K_I_s in the range of 52.5 nM – 1.81 µM), whereas Compounds **11**–**20** were not inhibitory (K_I_s > 10 µM). The best inhibitors were Compounds **5**–**9** (K_I_s in the range of 52.5 nM – 64.2 nM) and they incorporate nitro, chloro, and 2,5-dimethylphenyl moieties. The position of the R group on the phenyl moiety is crucial, since isomers such as Compounds **4** and **5/6** differ by an order of magnitude in their inhibitory action (Table 2). The 4-chloroderivative (Compound **4**) is roughly 10 times a weaker VchCAβ inhibitor compared to the 2- or 3-chlorosubstituted isomers (Compounds **5** and **6)**. Compounds **1**–**4** were medium potency inhibitors. It should be stressed that many of these *N*-hydroxyureas were more effective VchCAβ inhibitors compared to acetazolamide (Table 1), such as for example Compounds **3** and **5**–**9**.
- VchCAγ was generally poorly inhibited by most of the investigated *N*-hydroxyureas, except for Compounds **8, 9, 13, 14, 18**, and **19**, which were weak, micromolar inhibitors, K_I_s of 4.75 – 8.87 µM. The remaining 14 derivatives in the series were not inhibitory at all up to 10 µM concentration of inhibitor in the assay system (Table 1). It is in fact know that the active site of γ-CAs is rather shallow compared to the deep ones of the α- and β-class enzymes^3^.
- PgiCAβ was not significantly inhibited by any of the *N*-hydroxyureas Compounds **1–20** investigated here, which is rather difficult to explain considering the fact that the X-ray crystal structure of this enzyme is unknown. AAZ is on the other hand a medium potency inhibitor of this enzyme, with a K_I_ of 214 nM.
- The γ-CA from the same pathogenic bacterium, PgiCAγ, was on the other hand sensitive to inhibition by many of the investigated *N*-hydroxyureas Compounds **1–20**, which showed K_I_s ranging between 59.8 nM and 6.42 µM (Table 1). The best inhibitors were Compounds **8, 9,** and **14**, with K_I_s ranging between 59.8 and 84.4 nM. Again they contain nitrophenyl (Compounds **8** and **9**) and methylthiol-phenyl (Compound **14**) moieties in their molecule, which seem to be the best ones inducing an effective inhibitory activity against this enzyme. These three compounds were also much more effective than acetazolamide as PgiCAγ inhibitors.
- TweCAδ was poorly inhibited by Compounds **1–11**, whereas Compounds **12–20** showed a more effective inhibitory activity, with K_I_s of 33.3 nM – 8.74 µM (Table 1). The best inhibitors were Compounds **12–14**, with K_I_s of 33.3 – 57.8 nM and they incorporate various R moieties on the aryl fragment (3-methylthio, 2-ethoxy, and 2,5-dimethylphenyl). As for the other enzymes investigated here, the nature of the R moiety and substitution pattern on the aryl fragment are the main factors influencing he biological activity.
- The inhibition profile of these six CAs is very different between each other and also considering the human isoforms investigated earlier (hCA I, II, IX and XII)^14^, making this class of CAIs of particular interest for developing class-selective inhibitors.

## 4. Conclusions

A series of 20 *N’*-aryl-*N*-hydroxyureas possessing a variety of substitution patterns on the aryl fragment of the molecule, was investigated for the inhibition of six CAs belonging to four genetic families, from pathogenic bacteria and nonpathogenic diatoms. The α-/β-CAs from *V. cholerae* (VchCAα and VchCAβ) were effectively inhibited by some of these derivatives, with K_I_s in the range of 97.5 nM – 7.26 µM and 52.5 nM – 1.81 µM, respectively, whereas the γ-class enzyme VchCAγ was less sensitive to inhibition (K_I_s of 4.75 – 8.87 µM). The β-CA from the pathogenic bacterium *Porphyromonas gingivalis* (PgiCAβ) was not inhibited by these compounds (K_I_s > 10 µM) whereas the corresponding γ-class enzyme (PgiCAγ) was effectively inhibited (K_I_s of 59.8 nM – 6.42 µM). The δ-CA from the diatom *Thalassiosira weissflogii* (TweCAδ) showed effective inhibition with these derivatives (K_I_s of 33.3 nM – 8.74 µM). As most of these *N*-hydroxyureas are also ineffective as inhibitors of the human (h) widespread isoforms hCA I and II (K_I_s > 10 µM), this class of derivatives may lead to the development of CA inhibitors selective for bacterial/diatom enzymes over their human counterparts and thus to anti-infectives or agents with environmental applications.
